# SATB2 promotes humeral fracture healing in rats by activating the PI3K/AKT pathway

**DOI:** 10.1515/biol-2025-1126

**Published:** 2025-08-08

**Authors:** Liantao Liu, Shuai Rong, Xiaobin Zhou, Hao Li, Kepei Zhen, Chong Zheng, Kewei Li

**Affiliations:** Department of Pediatric Orthopedics, The Third Hospital of Shijiazhuang, No.15, South Sports Street, Chang’an District, Shijiazhuang, Hebei, 050011, China; Department of Trauma Orthopedics III, The Third Hospital of Shijiazhuang, Shijiazhuang, Hebei, 050011, China

**Keywords:** SATB2, PI3K/AKT, humeral fracture

## Abstract

The purpose of this study was to explore the potential mechanism of SATB2 and phosphatidylinositol 3-kinase/protein kinase B (PI3K/AKT) pathway promoting fracture healing *in vivo*. An SD model of humeral fracture in rats was established and treated. Following a 6-week treatment period, the morphology of the fracture was assessed. Serum interleukin 6 (IL-6), tumor necrosis factor-α (TNF-α), osteocalcin, C-telopeptide of type I collagen (CTX-I), and bone morphogenetic protein 2 (BMP-2) were determined. Alkaline phosphatase (ALP), receptor activator of nuclear factor-kappa B ligand (RANKL), osteoprotegerin (OPG), and other relevant molecules such as PI3K and p-AKT were measured. The results showed that SATB2 overexpression repaired humeral fracture and bone continuity. SATB2 overexpression resulted in a significant reduction in RANKL, IL-6, TNF-α, and CTX-I expression, while simultaneously increasing OPG, ALP, osteocalcin, and BMP-2. This indicates that SATB2 inhibits osteoclast activity and promotes osteoblast function. Additionally, SATB2 overexpression increased PI3K and p-AKT protein expression in humerus. Furthermore, the inhibitory effect of the PI3K/AKT inhibitor on PI3K and p-AKT protein expression was counterbalanced by upregulating SATB2. In conclusion, SATB2 promotes fracture healing in humeral fracture rats by stimulating the proliferation and differentiation of osteoblasts, which is related to the activation of PI3K/AKT signaling pathway.

## 1 Introduction

Fracture is the most common traumatic injury that occurs in the human body [1]. Despite the inherent ability of bone to self-repair, a significant proportion of patients, ranging from 10 to 20%, experience bone non-union or delayed healing [2]. Therefore, there is an urgent need to accelerate the development of treatment strategies aimed at enhancing the process of bone regeneration during fracture healing.

A highly conserved gene, special AT-rich sequence-binding protein 2 (SATB2) encodes a transcription factor involved in bone, craniofacial, and nervous system development [3,4]. The transcription factor SATB2 plays a key role in the correct facial pattern and normal bone development, and its absence in mice has been observed to diminish bone mineralization, ultimately leading to shortened and fragile bones in all limbs. This defect is attributed to increased expression of specific members of the Hox gene cluster and decreased expression of osteoblast-specific genes [5]. It has been found that SATB2 acts as a molecular regulator in the transcriptional network and coordinates the expression of some osteogenic transcription factors and matrix proteins, thus playing an important role in osteoblastogenesis [6,7]. Studies have shown that SATB2 regulates the genesis by regulating the expression and function of osteoblast-specific genes, such as interacting with Runt-related transcription factor 2 and activating transcription factor 4 and enhancing their transcriptional activities, affecting the process of bone formation, osteoblast differentiation, and matrix formation [4,8,9]. A known role of SATB2 is to regulate osterix, a transcription factor that regulates mesenchymal cells into osteoblasts. Additionally, it is thought to be involved in bone remodeling [10]. In summary, SATB2 can be a powerful osteoinductive molecule that recruits other transcription factors to form a platform or act as a molecular regulator of the transcriptional network. It can synergize and amplify, thereby multiplying the activity of multiple osteogenic transcription factors to regulate bone development and osteoblast differentiation. Although SATB2 can induce bone regeneration, whether it can play a key role in humeral fractures and help healing still needs to be explored in depth.

Protein kinase B (AKT) was initially identified as a proto-oncogene, serving as a signaling pathway of considerable significance in the context of post-traumatic repair and healing, as well as functioning as a regulatory pathway mediating cellular growth [11]. Phosphoinositide 3-kinase (PI3K) is activated by a variety of growth factors through receptor binding. The resulting products then interact with AKT, leading to AKT phosphorylation and subsequent modulation of the downstream target proteins Bad and Caspase. Ultimately, this regulatory mechanism controls cellular proliferation and differentiation [12]. PI3K/AKT axis is a regulator of bone metabolism that promotes bone formation and differentiation of immature osteoblasts into mature ones [13–15]. Dexamethasone (Dex) exerts a dual inhibitory effect on the phosphorylation of AKT and levels of semaphorin 3A (Sema3A) in bone marrow mesenchymal stem cells (BMSCs). Furthermore, Dex hinders osteoblast differentiation by suppressing Sema3A via the PI3K/AKT pathway [16]. PI3K/AKT signaling pathway is involved in the regulation of osteogenic precursor cell differentiation and activity [17]. The overall increase in PI3K signaling improves femoral regeneration by stimulating the proliferation of periosteal cells in the early stage of fracture healing. These studies have shown that the PI3K/AKT pathway is involved in bone metabolism and remodeling. Other studies have shown that inhibiting endothelial cell dysfunction by activating the PI3K/AKT pathway can reduce the age-related deterioration of angiogenesis at the fracture site, leading to increased angiogenesis and ultimately accelerating fracture healing [18,19]. The interaction between SATB2 and PI3K/AKT signaling pathway and its regulatory mechanism on humeral fracture healing still need to be further explored.

The process of fracture healing is a multifaceted biological phenomenon, wherein bone regeneration is significantly influenced by osteoblasts and osteoclasts. This research aims to investigate the potential mechanism underlying fracture healing *in vivo*, with a particular focus on the SATB2 and PI3K/AKT pathways. To achieve this, an animal model of humeral fracture was established.

## 2 Materials and methods

### 2.1 Humeral fracture animal model

A total of 60 male Sprague-Dawley rats, weighing 180 ± 20 g and aged 6 weeks, were procured from The Third Hospital of Shijiazhuang. The rats were housed in a facility with a regulated 12 h light–dark cycle and were given *ad libitum* access to food and water. The study was conducted with the approval of The Third Hospital of Shijiazhuang (No. 202106HB84) and adhered to the ethical principles outlined in the Guidelines for the Care and Use of Laboratory Animals.

For anesthesia, a 10% chloral hydrate solution (Sigma, USA) was administered via intraperitoneal injection. The humeral shaft, as well as the internal and lateral condyles of the humerus, was fully exposed. Subsequently, a 1 mm Kirschner needle was retrogradely drilled upward into the humeral bone marrow cavity. After penetrating the skin, any excess portion of the needle was removed by cutting it off. The upper one-third of the humerus was intentionally truncated using bone forceps, resulting in transverse fractures. Following wound cleansing, a dose of 40 U gentamicin (Sigma, USA) was administered into the wound, while 200,000 U of penicillin (Sigma) was injected intraperitoneally.


**Ethical approval:** The research related to animal use has been complied with all the relevant national regulations and institutional policies for the care and use of animals, and has been approved by the Third Hospital of Shijiazhuang Animal Experimental Ethics Committee (No. 202106HB84).

### 2.2 Treatment of fracture models

The rats were randomly divided into four groups: model group, SATB2 overexpression group, PI3K/AKT inhibition group, and SATB2 + PI3K/AKT inhibition group. Model group: humeral fracture + AAV5-GEP; SATB2 overexpression group: humeral fracture + AAV5-SATB2; PI3K/AKT inhibition group: humeral fracture + AAV5-GEP + PI3K/AKT-in-1; and SATB2 + PI3K/AKT inhibition group: humeral fracture + AAV5-SATB2 + PI3K/AKT-in-1.

To overexpress SATB2, an adenovirus encoding the SATB2 gene (AAV5-SATB2; HanBio, Shanghai, China) was injected into the tail vein of rats. AAV5-green fluorescent protein (HanBio) was used as a positive control (AAV5-GEP).

The rats were fasted for 24 h. The PI3K/AKT inhibition group and the SATB2 + PI3K/AKT inhibition group were administered with a PI3K/AKT inhibitor (PI3K/AKT-in-1, #HY-144806, MedChemExpress, USA) at 2 g/kg. The control group received an equivalent volume of normal saline.

### 2.3 Biomechanical test

At 3 and 6 weeks post-surgery, three rats were randomly chosen to undergo removal of the internal fixation (Kirschner needle) from the right humerus and bilateral fixation of the fractured humerus. The biomechanical characteristics were assessed using a three-point bending test, utilizing a mechanical testing apparatus with a 20 mm span and a loading rate of 1 mm/min. The maximum load and stiffness were recorded, with the callus serving as the point of loading.

### 2.4 Enzyme-linked immunosorbent assay (ELISA)

At 6 weeks after surgery, six rats were chosen from each group and their abdominal venous blood was extracted and subjected to centrifugation to collect the supernatant. Interleukin 6 (IL-6), tumor necrosis factor (TNF)-α, osteocalcin, C-telopeptide-type-I-collagen (CTX-I), and bone morphogenetic protein-2 (BMP-2) in the rat serum or plasma were determined using ELISA kits (R&D Systems, USA). Using a microplate reader, absorbance values were measured at 450 nm. NO level (Griess method and NO assay kit; Jiancheng Biological, Nanjing, China) and eNOS activity (Rat Endothelial NOS (eNOS) ELISA Kit; Jinln Biological) in rat plasma were determined.

### 2.5 Immunohistochemistry (IHC)

The tissue sections were treated with citrate buffer at 60°C. Subsequently, the slices underwent an 8 min incubation with protease K to facilitate antigen retrieval. Following this, the slices were incubated overnight at 4°C with PI3K (#4228; Cell Signaling Technology, USA), P-AKT (#4060; Cell Signaling Technology), and alkaline phosphatase (ALP; #ab83259, Abcam, USA). After incubation with the secondary antibody immunoglobulin G (#ab124055; Abcam) at room temperature for 1 h, the target signal was activated using 3,3′-diaminobenzidine substrate (Vector Labs, CA, USA). Finally, the slides were re-stained with hematoxylin for 2 min and subsequently observed. A semi-quantitative technique was employed for immunopositive assessments [20]. For each microscopic field, cells were counted as either immunopositive or negative and then expressed as percentages. Positive cell percentage was evaluated on a 5-point scale: 0 for no positive cells, 1 for 20%, 2 for 21–50%, 3 for 51–70%, and 4 for 71%. Immunostaining intensity was evaluated using a 4-level scoring system: 0 for no staining, 1 for low intensity, 2 for moderate intensity, and 3 for high intensity. The IHC score was calculated by multiplying the scores for cell positivity and signal intensity, ranging from a minimum of 0 to a maximum of 12.

### 2.6 Imaging analysis

Following the successful establishment of the fracture model and drug injection, lateral femoral radiographs were taken at 1, 3, and 6 weeks using specific parameters (52 kV, 4.5 mAs, 0.5 s). These radiographs were utilized to observe the growth of bone callus, the position of the internal fixation, the healing of the fracture line, and the alignment of the fracture line by X-ray film analysis. Each X-ray was independently reviewed by a radiologist, a lab researcher, and a plastic surgeon. Subsequently, the rats were euthanized, and the callus was extracted from the cadaver and placed in liquid nitrogen.

### 2.7 Hematoxylin and eosin (HE) staining

The skin incision was made and the muscles were promptly dissected. Subsequently, the humerus was immobilized in 10% formaldehyde solution (Beyotime, Shanghai, China), decalcified in 9% formic acid solution (Beyotime), and embedded in paraffin. Following standardized processing, the specimens were sectioned longitudinally at 5 μm thickness, embedded in paraffin, and subjected to staining with hematoxylin solution, followed by eosin solution. Imaging was conducted using a microscope (DS-Ri 2, Nikon, Japan).

### 2.8 Masson (MS) dyeing

The prepared paraffin sections were stained with Regaud’s Hematoxylin staining solution, then stained with Lichun red acid fuchsin dye (Sinopharm, Shanghai), and finally treated with aniline blue. New or mature bones were observed under a microscope.

### 2.9 Real-time reverse transcriptase-polymerase chain reaction (RT-qPCR)

RNA was extracted from the fracture site using TRIzol® reagents (Invitrogen, CA, USA). The RNA quality was then measured by Nanodrop 2000. cDNA samples were obtained using TaKaRa RNA PCR kit (TaKaRa, Dalian, China) and Oligo dT primer (Invitrogen). Gene expression levels were detected using SYBR mixture (TaKaRa, Dalian, China) on a LightCycler 480 device (Roche, Basel, Switzerland). Primer design is shown in Table 1. Gene expression was normalized to glyceraldehyde-3-phosphate dehydrogenase (GAPDH) and the data were analyzed using the 2^−ΔΔCT^ method.

**Table 1:** Primers

Name	Primer sequences (5′–3′)
SATB2	TAAGCAGTCCCTGCGCGTTT
	GAATCATCAGACCTCCCACGG
GAPDH	CATCATCCCTGCCTCTACTGG
	GTGGGTGTCGCTGTGTGAAGTC

### 2.10 Western blotting analysis

Protein was extracted from the fracture site, which included the entire callus and adjacent bone less than 2 mm. Following the full protein extraction kit’s guidelines, the lysis buffer was introduced, blended in a tissue homogenizer for 1 min, and then spun at 12,000 rpm and 4°C for 15 min. After gathering the supernatant, the protein levels were measured using the bicinchoninic acid protein quantification kit (Pierce, MA, USA). The proteins were separated by 15% sodium dodecyl sulfate-polyacrylamide gel electrophoresis, transferred to polyvinylidene difluoride (Beyotime) membranes, blocked at room temperature with 5% skim milk powder for 1 h, and washed three times with TBST (Vazyme, Nanjing, China). PI3K (#4228; Cell Signaling Technology), p-AKT (#4060; Cell Signaling Technology), AKT (#9272; Cell Signaling Technology), p65 (#10745-1-AP; Proteintech), p-p65 (#3033; Cell Signaling Technology), ALP (#ab83259; Abcam), receptor activator of nuclear factor-kappa B ligand (RANKL; #ab45039, Abcam), osteoprotegerin (OPG; #ab73400, Abcam), and GAPDH (#ab246513; Abcam) were incubated overnight at 4°C. After three washing of TBST, the secondary antibody (Cell Signaling Technology) bound to the corresponding horseradish peroxidase was incubated at 37°C for 1 h and developed using an enhanced chemiluminescence kit (Sigma).

### 2.11 Statistical analysis

**Figure 1:**
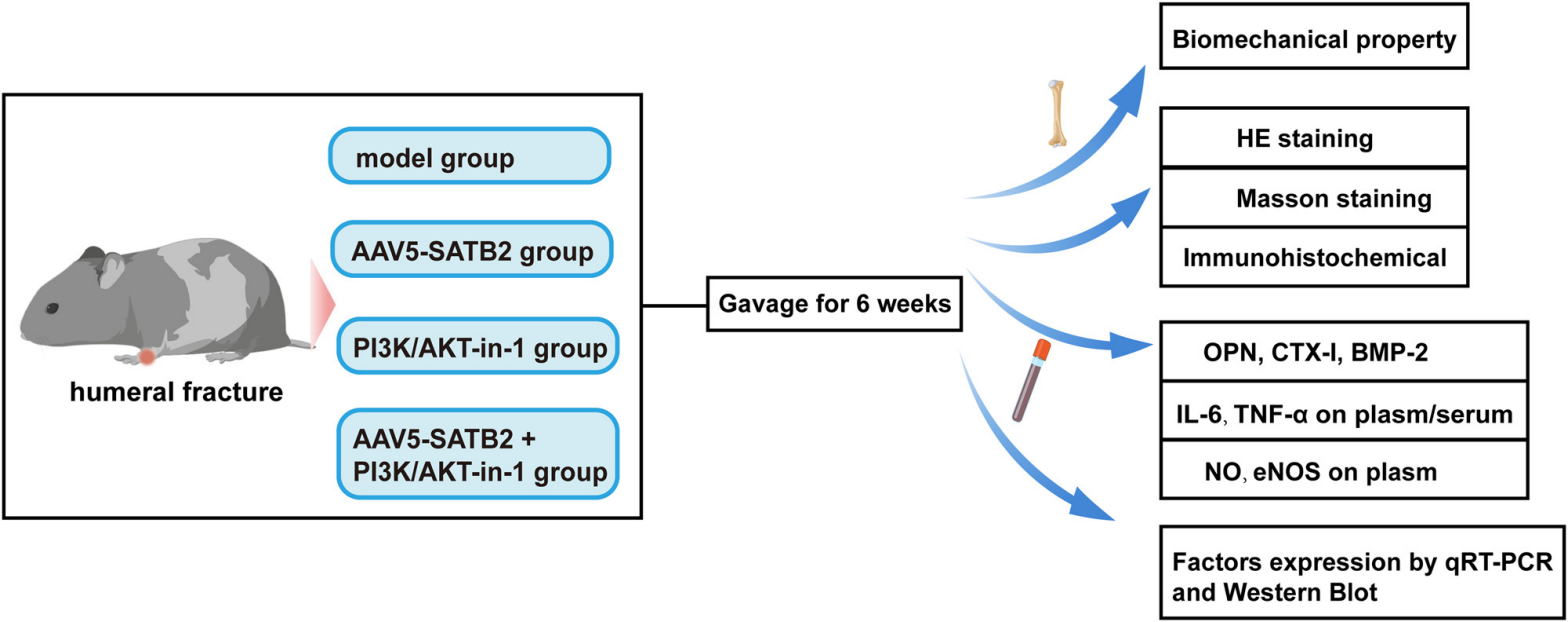
Experimental design. Stable humeral fractures were established in SD rats. Biomechanical properties were assessed by three-point bending test at postoperative weeks 3 and 6. Tissue sections were prepared and subjected to HE staining and Masson staining to assess osteogenic differentiation. IHC assay was performed to assess the expression of ALP and PI3K/Akt pathway-related proteins. The expression of SATB2 and its related factors was quantified by RT-qPCR, ELISA, and western blot.

The experimental design is shown in Figure 1. Statistical analysis was conducted using GraphPad Prism 8 (GraphPad Software, USA) and SPSS Statistics 20 (IBM, USA). The data were presented as mean ± standard deviation. All experiments were replicated biologically at least three times. Student’s *t*-test was employed to assess differences between the two groups, while one-way analysis of variance was used for multiple recombination comparisons. A significance level of *p < 0.05 was deemed statistically significant.

## 3 Results

### 3.1 SATB2 promotes humeral fracture healing

**Figure 2:**
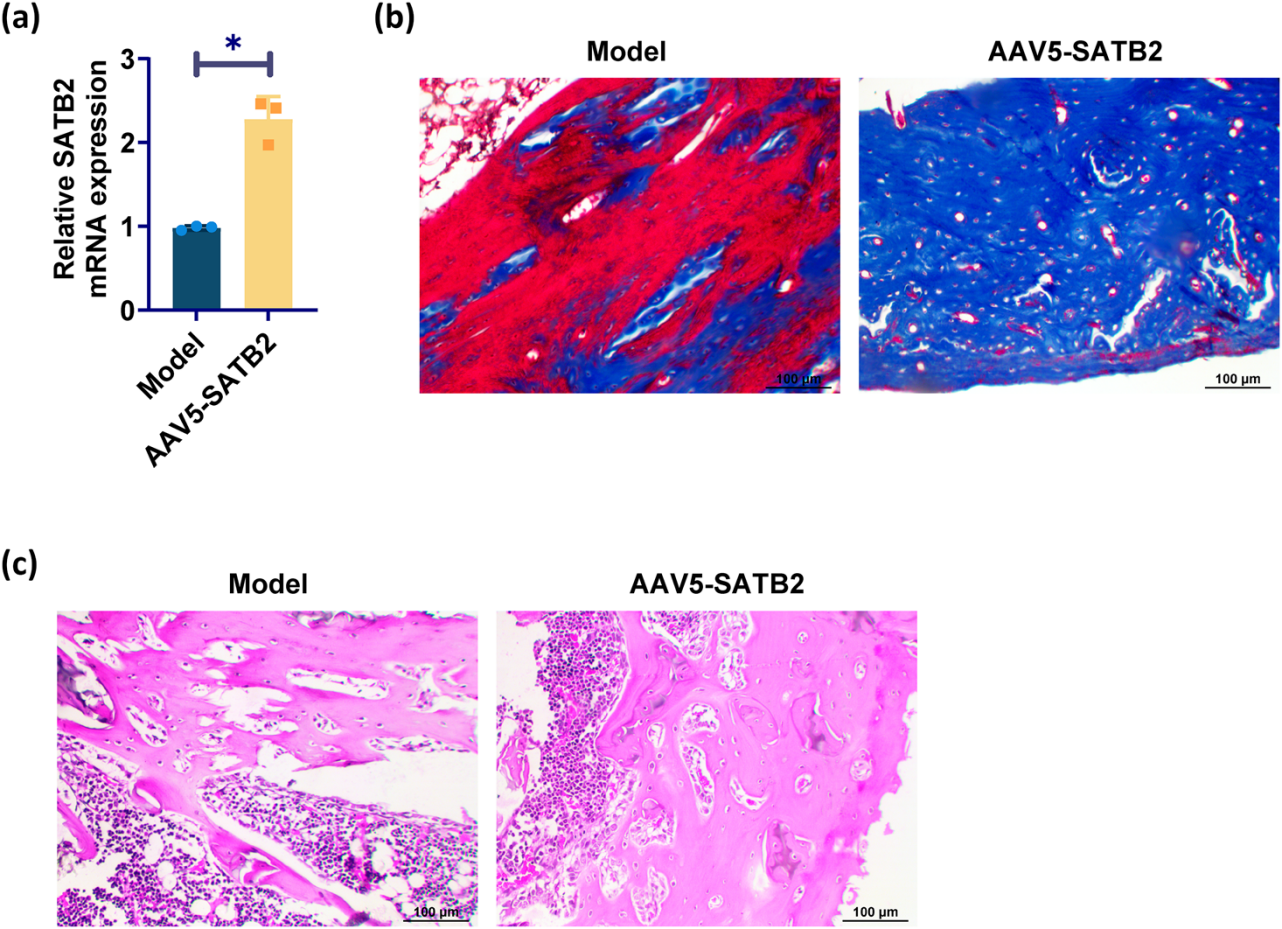
SATB2 promotes humeral fracture healing: (a) RT-qPCR was used to detect SATB2 expression, (b) MS staining shows the degree of fracture healing, and (c) HE staining shows histological findings of bone healing. *p < 0.05 was considered statistically significant.

RT-qPCR analysis demonstrated a significant upregulation of SATB2 expression in the humerus of the SATB2 overexpression group compared to the model group (Figure 2a). Histological analysis using MS staining at week 6 revealed a greater extent of bone formation, improved bone quality, and increased blue staining indicative of favorable bone repair in the SATB2 overexpression group as compared to the model group. SATB2 overexpression promoted the healing of humeral fractures in rats based on these findings (Figure 2b). Further observation by HE staining showed that the fracture end of the SATB2 overexpression group was significantly repaired and the bone continuity was restored. In the model group, the bone repair of the fracture end was not obvious, and the continuity of the fracture end was poor (Figure 2c). As shown in Table 2, the maximum load, stiffness, and total energy absorption of fracture callus in the SATB2 overexpression group were significantly higher than those in the model group.

**Table 2:** Comparison of biomechanics of fracture callus between the two groups

Time	Groups	Maximum load (N)	Stiffness (N/mm)	Total energy absorption (mJ)
Three weeks after fracture	Model group	22.1 ± 4.5	101.6 ± 13.5	13.7 ± 3.7
	SATB2 overexpression group	52.3 ± 9.8*	226.8 ± 26.4*	34.1 ± 5.7*
Six weeks after fracture	Model group	75.9 ± 10.2	158.4 ± 20.1	23.6 ± 4.2
	SATB2 overexpression group	142.1 ± 19.8*	267.3 ± 30.9*	46.2 ± 8.10*

*p < 0.05 indicates statistical significance of the difference.

### 3.2 Overexpression of SATB2 enhances osteogenic differentiation

**Figure 3:**
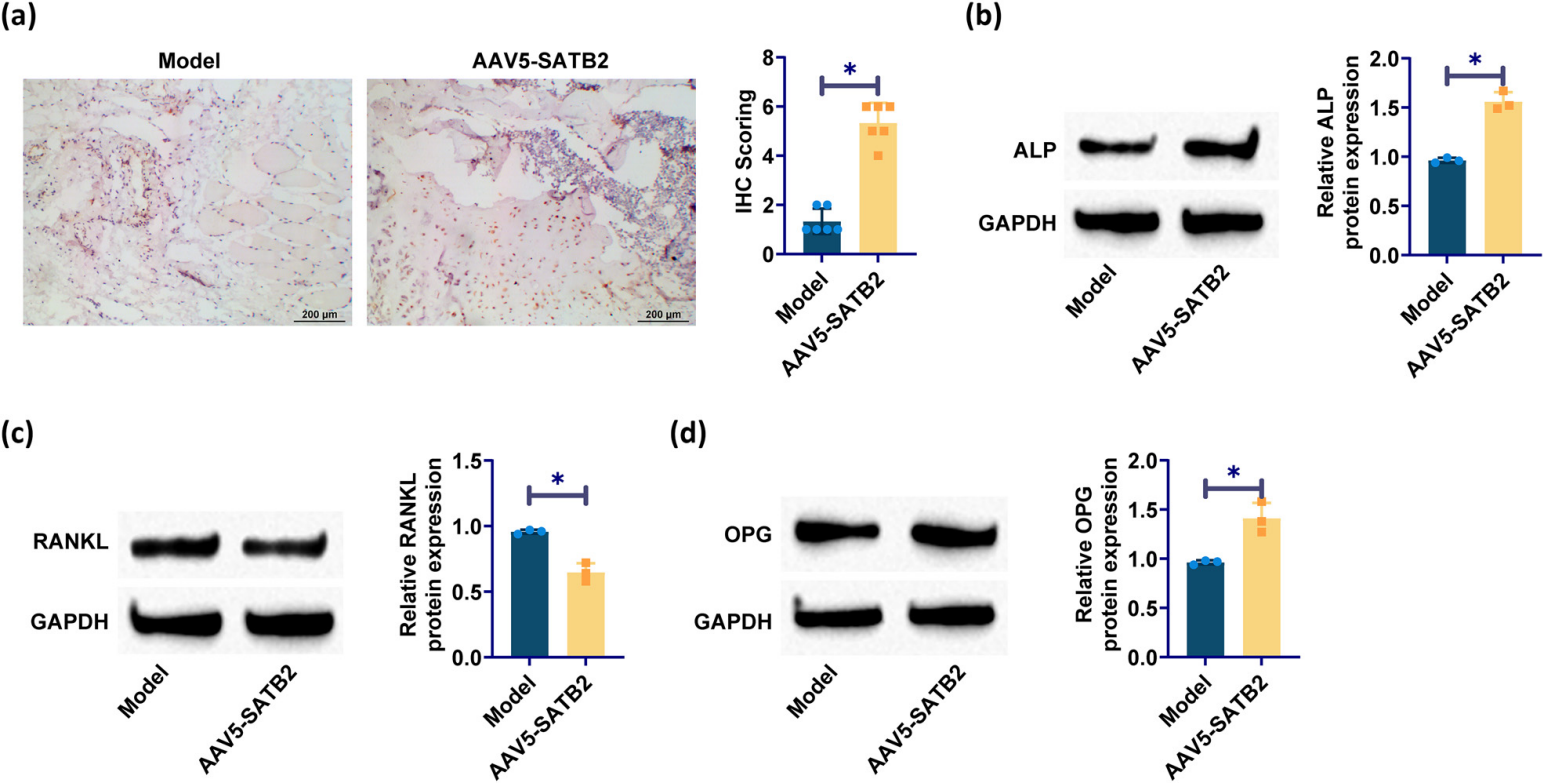
Overexpression of SATB2 enhances osteogenic differentiation: (a) IHC staining detected ALP; (b)–(d) western blotting detection of ALP, RANKL, and OPG. *p < 0.05 was considered statistically significant.

To further elucidate the effect of SATB2 overexpression on osteogenic differentiation, western blotting was used to identify the presence of osteogenic markers such as ALP, RANKL, and OPG. The signaling pathways involving ALP, OPG, and RANKL are known to exert significant regulatory control over the process of osteoclast differentiation and maturation. The upregulation of RANKL can induce the transformation of osteoblasts into osteoclasts, thereby enhancing osteoclast activity and promoting bone resorption, ultimately culminating in fracture nonunion. IHC staining showed that the number of ALP-positive cells in the SATB2 overexpression group was significantly increased, which was confirmed by western blotting (Figure 3a and b). Compared with the model group, the SATB2 overexpression group exhibited significantly decreased RANKL expression and increased OPG, indicating that SATB2 overexpression inhibited osteoclast activity and promoted osteoblast function (Figure 3c and d).

### 3.3 SATB2 affects serum osteocalcin, CTX-I, BMP-2, and inflammation

**Figure 4:**
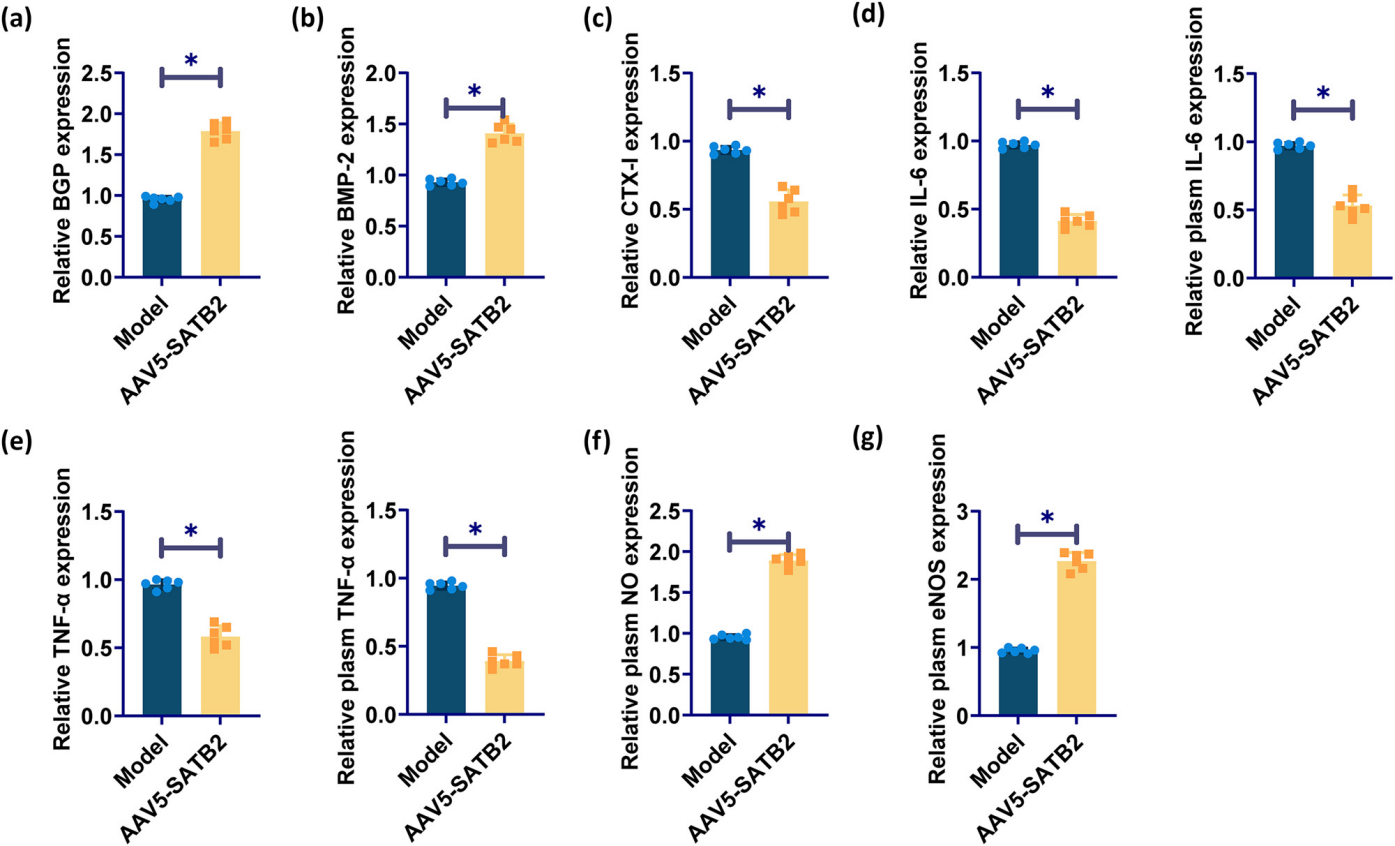
Effects of SATB2 on serum osteocalcin, CTX-I, BMP-2, and inflammation. (a)–(c) ELISA detected serum osteocalcin, BMP-2, and CTX-I in rats. (d) ELISA for the determination of IL-6 in rat plasma and serum. (e) ELISA for the detection of TNF-α in rat plasma and serum. (f) and (g) ELISA for plasma NO level and eNOS activity in rats. *p < 0.05 was considered statistically significant.

Osteocalcin and BMP-2 in the serum of model group was lower, and CTX-I was higher. Compared with the model group, osteocalcin and BMP-2 in the SATB2 overexpression group were increased, and CTX-I was decreased (Figure 4a–c). The expression of inflammatory factors IL-6 and TNF-α was higher in the plasma and serum of rats in the model group, which was significantly reduced by SATB2 overexpression (Figure 4d and e). NO levels and eNOS activity were also detected in the plasma of rats. The plasma levels of NO and eNOS were significantly up-regulated under the effect of SATB2 overexpression (Figure 4f and g).

### 3.4 Expression of PI3K/AKT signaling pathway-related proteins in humerus tissues of rats

**Figure 5:**
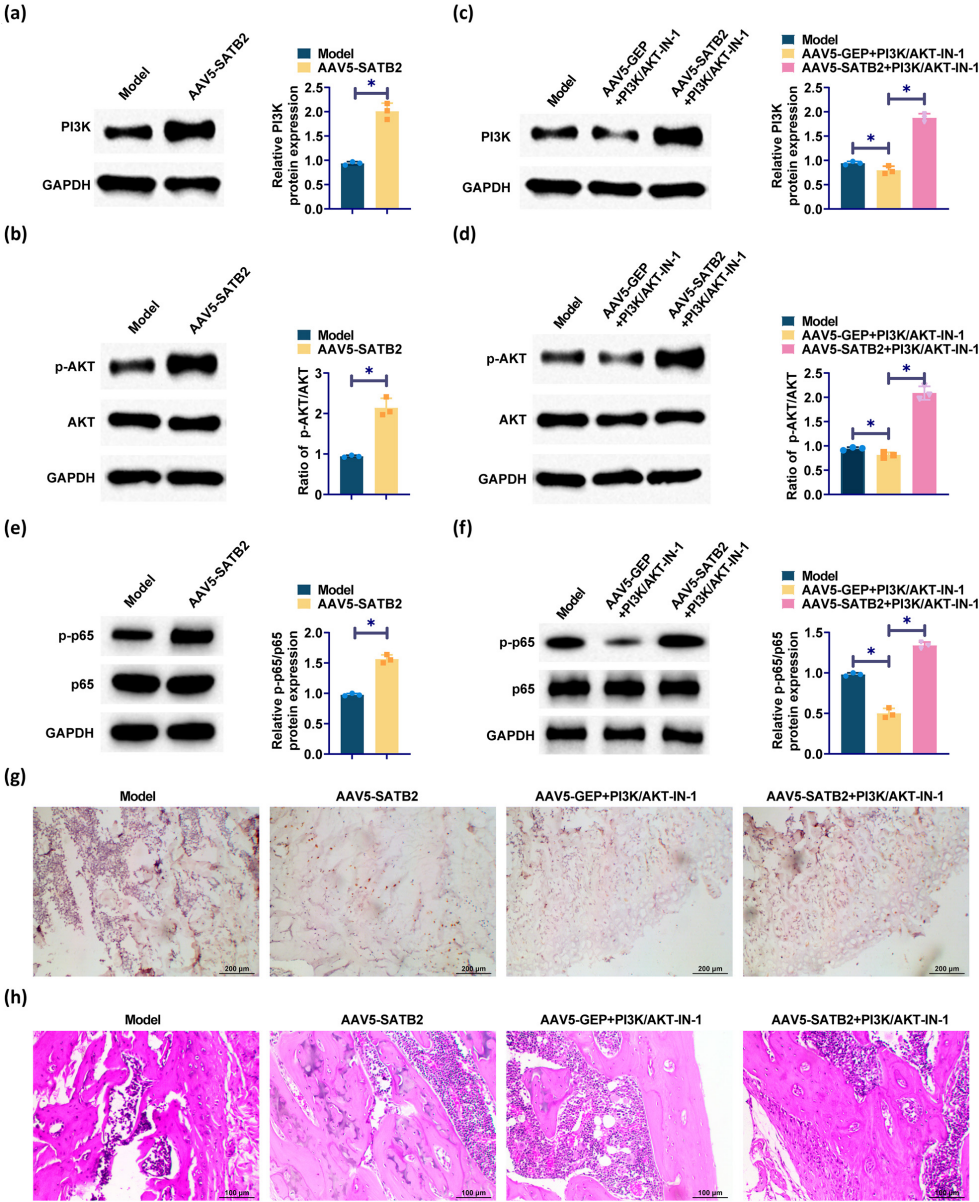
Expression of PI3K/AKT pathway-related proteins in humerus tissues of rats. (a) and (c) Western blotting detected PI3K expression. (b) and (d) Western blotting detected p-AKT expression. (e) and (f) Western bolt for p-65 and p-p65 expression. (g) Immunohistochemical staining to detect the expression of PI3K and AKT in the fracture region of rats in each group. (h) HE staining showed histological findings of bone healing. *p < 0.05 was considered statistically significant.

To detect whether the PI3K/AKT pathway is expressed and activated during the whole healing process of fractured rats, western blotting was conducted to detect key proteins. Compared with the model group, PI3K and p-AKT in the humerus in the SATB2 overexpression group was significantly increased, as well as p-p65/p65 (Figure 5a, b, and e). SATB2 indeed activated the PI3K/AKT signaling pathway. It is worth noting that PI3K/AKT and p-p65/p65 expression was inhibited by PI3K/AKT inhibitor. However, upon overexpression of SATB2, the reduction in PI3K and p-AKT proteins induced by PI3K/AKT inhibitor was reversed (Figure 5c, d, and f). SATB2 affected the PI3K/AKT signaling pathway to a greater extent. IHC staining results showed that compared with the model group and the PI3K/AKT inhibition group, the positive rate of PI3K and p-AKT in the fracture area of rats after SATB2 overexpression also confirmed that SATB2 can activate the PI3K/AKT pathway (Figure 5g). HE staining also showed poor healing and repair in the PI3K/AKT inhibition group, but the healing and repair difference caused by PI3K/AKT inhibitor was reversed after overexpression of SATB2 (Figure 5h).

## 4 Discussion

In recent times, there has been a significant focus on the comprehensive investigation of the molecular mechanisms underlying the process of fracture healing. In particular, the protein SATB2 has emerged as a key factor in the complex regulation of osteogenic differentiation and osteoblast formation [3,21]. This study investigated the potential therapeutic effect of SATB2 on fracture healing in a rat model of humeral fracture. In the SATB2 overexpression group, bone repair was evident and bone continuity was restored. In the model group, bone repair was not evident and fracture continuity was poor.

RANK is a specific receptor of RANKL, and both recognize and bind on the surface of osteoclasts, which can promote differentiation and maturation of osteoclasts and inhibit their apoptosis, thus leading to bone resorption and reducing bone density [22]. OPG is primarily secreted by osteoblasts and BMSCs, exerting a pivotal function in the suppression of bone resorption and the enhancement of bone density [23–25]. In this experiment, it was found that OPG protein was lower in the bone tissue of the model group, while RANKL was higher. During fracture repair, osteoblasts and vascular bone endothelium can synthesize ALP, and the secreted ALP can penetrate into the blood, increasing the blood levels of ALP [26]. In this study, ALP levels in the SATB2 overexpression group were significantly higher than those in the model group.

Osteocalcin, a polypeptide, is produced and released by bone cells in response to 1,25-(OH)2D3 stimulation. Its primary function is to impede the development of atypical hydroxyapatite crystals and uphold the customary rate of bone mineralization. The measurement of serum osteocalcin serves as an indicator of recent osteocalcin synthesis and bone formation [27]. BMP-2 regulates the proliferation and differentiation of cells. As a factor in promoting bone formation, BMP-2 plays a decisive role in the differentiation of osteoblasts [28]. Changes in CTX-I during fracture healing are more sensitive than changes in bone mineral density and can therefore be used to assess bone metabolism and bone resorption [29]. The inflammatory response is a significant contributor to the delayed healing process of fractures. The upregulation of IL-6, TNF-α, and other inflammatory mediators can lead to the degradation of the extracellular matrix of chondrocytes, thereby impeding chondrocyte differentiation and resulting in a decelerated fracture healing process [30,31]. This study showed that BMP-2 and osteocalcin in the serum of rats in the SATB2 overexpression group were increased, while CTX-I, IL-6, and TNF-α were decreased. These results suggest that SATB2 can promote osteoblast proliferation and differentiation and inhibit osteoclast activity to promote fracture healing.

In the PI3K/AKT signaling pathway, AKT serves as an essential protein, functioning as a primary kinase downstream of PI3K and contributing to cell survival, growth, and proliferation. The activation of AKT is mediated by p-AKT, which then activates mTOR, a component downstream of the PI3K/AKT signaling pathway, thus impacting life activities such as apoptosis. The role of the PI3K/AKT signaling pathway in bone formation and regeneration is considered important. Within the PI3K/AKT pathway, the regulatory role of PI3K signaling is significant in driving BMSCs to develop into osteoblasts and aid in bone formation [32]. MiR-21 promotes fracture healing in rats by activating PI3K–AKT signaling pathway [33]. The inhibition of β-catenin transcription weakens the PI3K–AKT-induced proliferation, differentiation, and mineralization of osteoblasts, highlighting the Wnt/PI3K/AKT/β-catenin signaling pathway in osteoblasts and implying a strong relationship between the PI3K–AKT pathway and fracture healing [34]. This is also consistent with our experimental results. Compared with the SATB2 overexpression group with fracture healing, PI3K and AKT were significantly decreased in the model group with poor fracture healing. PI3K/AKT pathway can promote fracture healing and may be a potential mechanism to promote fracture healing [35]. Western blotting detected lower PI3K and AKT in the PI3K/AKT inhibition, but PI3K/AKT expressions increased after SATB2 overexpression. We speculate that the mechanism of SATB2 promoting fracture healing may be to promote osteoblast function by inducing AKT phosphorylation and up-regulation of PI3K–AKT signaling pathway, thereby accelerating fracture healing. However, we still need to understand how these signaling pathways are related to each other to better understand the potential mechanism of SATB2’s effect on fracture healing.

Using a fracture rat model, we found that SATB2 significantly enhanced the regeneration of damaged bone tissue. For example, overexpression of SATB2 accelerated bone formation by positively regulating the expression of multiple osteoblast-specific genes and enhanced the increase of new bone formation in bone defects. The integration of SATB2 in bone tissue engineering offers novel and essential insights into gene detection, treatment, and molecular regulation of bone tissue regeneration, representing a promising alternative strategy to expedite bone regeneration. With further development, the use of SATB2 as a gene therapy for bone defects may shorten the healing time of human bone defects and become a valuable strategy to reduce recovery time and external fixation requirements.

Previous studies have shown that SATB2 is a high-order transcription factor that regulates genes required for osteogenic differentiation. A significant feature of this study is that in addition to its role in differentiation, it has also been found that SATB2 interacts with the PI3K/Akt signaling pathway to achieve dual regulation of osteoblast and osteoclast differentiation and maturation, and to regulate the expression of factors associated with the inflammatory response. The involvement of SATB2 in the differentiation and inflammatory response processes and the promotion of the biological properties of the fracture callus make SATB2 an interesting target for the treatment and healing of bone regeneration disorders. Although this finding is the advantage of this study, there are still some limitations. This study used only rat models and did not validate the role of SATB2 in combined old age or osteoporotic fracture models. Micro-CT scans were not performed, and the assessment of bone healing quality was incomplete. Second, we were unable to determine whether there is a direct interaction between SATB2 and the PI3K/Akt signaling pathway. Whether the upstream regulatory mechanism of eNOS/NO is dependent on the PI3K/Akt signaling pathway is also unknown. It is worth focusing on the fact that the current study found that SATB2 upregulation may be involved in tumorigenesis. Therefore it is necessary to evaluate it in future studies in the long term. We will attack these limitations one by one in the future. Extending the pathological state is also a direction we will further explore in the future. Meanwhile, knockdown of AKT or p65 using CRISPR is used to elucidate the precise regulation of key nodes by SATB2.

## 5 Conclusion

The present study systematically revealed the key role of transcription factor SATB2 and its molecular mechanism in promoting humerus fracture healing, which provides an important theoretical breakthrough and clinical translational prospect in the field of bone repair. SATB2 promotes the proliferation and differentiation of osteoblasts by activating the PI3K/Akt signaling pathway, and inhibits osteoclastogenic activity by regulating the balance of OPG/RANKL. Meanwhile, SATB2 improves local blood supply by up-regulating eNOS/NO and reduces the level of inflammatory factors such as IL-6/TNF-α. Compared to traditional single-pathway pro-healing strategies, SATB2 exhibits multi-target synergistic benefits that make it of significant translational medicine value. Although the role of SATB2 in pathological states such as osteoporosis and diabetes mellitus has not yet been validated, the present study provides a theoretical basis for SATB2 in the clinical treatment of fractures. Future studies will further validate the therapeutic effect of SATB2, clarify the applicable types of SATB2 in clinical practice, and further deepen the understanding of the mechanism of SATB2’s action on bone repair.
